# Robot-Assisted Tubal Reanastomosis after Sterilization: A Choice for Family Planning

**DOI:** 10.3390/jcm11154385

**Published:** 2022-07-28

**Authors:** Arwa Salehjawich, Veronika Günther, Zino Ruchay, Mazhar Salim Al Zoubi, Juhi Dhanawat, Nicolai Maass, Johannes Ackermann, Julian Pape, Ibrahim Alkatout

**Affiliations:** 1 Department of Obstetrics and Gynecology, University Hospitals Schleswig-Holstein, Campus Kiel, Arnold-Heller-Straße 3 (House C), 24105 Kiel, Germany; arwa.salehjawich@uksh.de (A.S.); veronika.guenther@uksh.de (V.G.); zino.ruchay@uksh.de (Z.R.); juhidhanawat@gmail.com (J.D.); nicolai.maass@uksh.de (N.M.); johannes.ackermann@uksh.de (J.A.); julianmaria.pape@uksh.de (J.P.); 2 Department of Basic Medical Sciences, Faculty of Medicine, Yarmouk University, Irbid 211-63, Jordan; mszoubi@yu.edu.jo

**Keywords:** family planning, sterilization, robot-assisted, regret, tubal reanastomosis

## Abstract

A variety of procedures have been used for family planning. One of these is sterilization surgery, which can be reversed by a tubal reanastomosis. In the present report, we compare Robot-assisted tubal reanastomosis sterilization with other methods of family planning and discuss factors related to the choice of the approach. The keywords used for the electronic search in PubMed were family planning, sterilization, Robot-assisted, tubal reanastomosis, depression, and regret. The decision in favor of or against sterilization surgery has been a sensitive issue for several years. Robot-assisted technology is a modern and precise approach. It has contributed to the flexibility of the decision between sterilization and its reversal through tubal reanastomosis, as well as enhanced the success rate of the surgery. Based on our analysis of the published literature, we believe that Robot-assisted tubal anastomosis is the optimum approach. However, to ensure the quality of health care, the surgeon must be well trained, well versed with the anatomy of the fallopian tubes, and thoroughly informed on the psychological impact of family planning.

## 1. Introduction

A rising number of contraceptive approaches are being used throughout the world. Currently, about 63% of women in the world are aged 15 to 49 years [1]. Due to modern lifestyles and career opportunities, contraception for women has emerged as a subject of great interest. The provision of effective and affordable contraception for all fertile women is one of the declared goals of the United Nations. Affordable contraception enables couples to make responsible decisions concerning reproduction; it also improves maternal and infant health by preventing inadvertent or closely-spaced gestations.

Sterilization, one of the most common methods of contraception for women, accounts for 30% (200 million) of all the contraception methods used by women. Notably, 5–20% of sterilized women regret their decision later in life and only 1–2% request a reversal of sterilization [1,2,3]. Factors associated with regret include sterilization at a young age or shortly after giving birth, a new relationship, and a low socioeconomic status. A relationship with a new partner is the most common reason for desiring a reversal of sterilization. In rare cases, the death of a child may cause a woman to request a reversal of tubal sterilization. The last few decades have witnessed the introduction of several procedures for the reversal of sterilization. Laparotomy, the first of these, was performed in the early 1970s [4,5] and was based on a midline abdominal incision through which the fallopian tubes could be accessed. The occluded ends of the tubes were excised, and methylene blue was instilled to test the patency of the tubes. The anastomosis was performed with sutures and strengthened with a splint. The technical principles have remained largely the same. A microscopic camera during surgery allowed for much greater accuracy and made it possible to perform intricate anastomoses of various types. The most frequently used approach was the two-layer technique, by which the muscular and serosal layers were sutured separately with varying numbers of stitches. Occasionally, a splint was used to bridge the lumen of the proximal and distal parts of the fallopian tube, and then removed subsequently [6]. The laparoscopic approach was introduced during the same period. These techniques were used for several years.

In the meantime, the quest for surgical techniques to enhance the quality of anastomosis continued [7,8]. The first Robot-assisted tubal reanastomosis, with closure in two layers, was performed with the ZEUS robotic system in 1998. The technique resulted in a patent anastomosis [6]. Two options are available to sterilized women who desire children: reversal of sterilization or IVF. Prior to obtaining informed consent, women must be informed about pregnancy rates after these two options. Furthermore, before undergoing surgical sterilization, women must be counseled extensively about other contraceptive measures.

## 2. Anatomy of the Fallopian Tube

**Figure 1:**
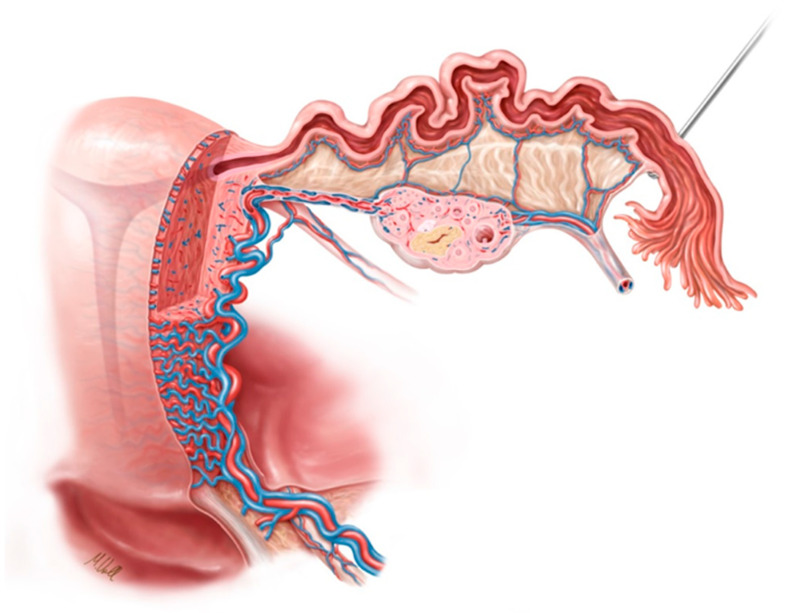
Anatomy of the fallopian tube with arterial and venous blood flow.

Figure 1 shows the anatomy of the fallopian tube with arterial and venous blood flow.

## 3. Blood Supply and Lymphatics

Arterial blood supply to the fallopian tubes originates at the anastomoses between the ovarian and tubal branches of the ovarian artery and the ascending branches of the uterine artery. The ovarian arteries are ramifications of the inferior abdominal aorta, just below the renal artery. Uterine arteries arise from the internal iliac arteries. The ascending branches move upward to the uterine horns and the descending branches proceed towards the superior vagina. The lateral fallopian tube is supplied by the ovarian arteries, and the medial fallopian tube by the ascending branches of the uterine artery. Ischemia in any portion of the tube is avoided by anastomoses between the two vessels. Analogous to arterial blood supply, venous blood flows from the fallopian tubes to the tubal branches of the uterine and ovarian veins. Blood flows from the right ovarian vein into the inferior vena cava (IVC), and from the left ovarian vein into the left renal vein. The uterine veins drain into the internal iliac veins, and the latter into the IVC. The lymphatics of the fallopian tubes follow a similar pattern as those of the ovaries. Lymph flows from the fallopian tubes to the para-aortic (lumbar) and pelvic lymph nodes [8,9].

## 4. Nerves

Afferent nerve fibers of the fallopian tubes follow the same route as sympathetic efferent nerves, which originate from T11, T12, and L1. By contrast, minor parasympathetic innervation to the lateral portion of the fallopian tube is shared between vagal fibers of the ovarian plexus, while the medial portion is innervated by the pelvic splanchnic nerve from the sacral spinal nerves S1, S2, and S3. The most-dense innervation exists in the medial isthmus [9].

## 5. Muscles

The fallopian tubes have a muscular layer covered by the inner mucous membrane (containing longitudinal cilia extending into the lumen) and the outermost serosa. The latter is also known as the muscularis mucosa, consisting of an inner circular layer and an outer longitudinal layer [8,9].

## 6. Family Planning

Sterilization, oral contraceptives, and condoms are the most commonly used methods of contraception in Western countries. Condoms possess the advantage of reducing the risk of STDs and cervical cancer. Of intrauterine devices (IUDs), the most commonly used ones are the copper T380A ParaGard^®^ and the levonorgestrel-releasing intrauterine system (IUS) Mirena^®^ or Kyleena^®^. When used correctly, further options such as the combined estrogen–progestin oral contraceptive, the patch, and vaginal rings provide reliable contraception [10,11]. Table 1 summarizes the existing contraceptive methods [12].

Methods of safe long-term contraception include laparoscopic bipolar electrocautery at three adjacent sites on each tube, the silastic band, or the Filshie clip. Hysteroscopic sterilization techniques may be used to achieve effective and permanent contraception in women, and do not require general anesthesia or an abdominal incision. Vasectomy is a highly effective and economical method of sterilization for men, with no risk of heart disease or prostate cancer [13,14,15,16,17].

## 7. Reasons for Performing Sterilization

Completed family planning and no desire for further children is the most common reason for surgical sterilization. Social and economic factors, health issues, situational factors (such as age or economic status), as well as encouragement by the family or physician may influence the decision. Some women have many reasons for undergoing sterilization. Tubal sterilizations are most commonly performed because a woman desires no further children. Women who wish to undergo sterilization for other reasons are more likely to regret the step later [18].

## 8. Index of Sterilization

The formula used at many centers (endorsed by the American College of Obstetricians and Gynecologists until 1969) is that elective sterilization may be considered only when age multiplied by parity is greater than or equal to 120.

## 9. Types of Sterilization

Two types of sterilization have been described: puerperal sterilization at the time of cesarean section, and sterilization performed several days after vaginal delivery. Non-puerperal sterilization, also known as interval sterilization, is not related to delivery. The procedure consists of occlusion or division of the fallopian tube, and can be performed by the laparoscopic or hysteroscopic approach or through a mini-laparotomy.

Commonly used procedures based on laparotomy include the Parkland, the Pomeroy, and the modified Pomeroy technique. Laparoscopic procedures of sterilization include unipolar or bipolar electrocoagulation, using the Falope ring, the Hulka clip, or the Filshie clip. Sterilization by transcervical hysteroscopy is achieved with the aid of the Essure or Adiana system, both of which are approved by the FDA.

Since electrocoagulation causes greater tissue damage than mechanical occlusion, the latter procedure is more feasible for the reversal of sterilization. Hysteroscopic sterilization with the Essure or Adiana system is irreversible.

## 10. Methods of Sterilization

***The Pomeroy technique:*** The base of the tube is ligated with a single absorbable suture, and a loop of the tube is excised. Alternatively, the mid-portion of the tube is excised after ligation of the segment with two absorbable sutures.

***The Irving technique:*** A central portion of the tube is excised. Both stumps are sutured to the uterus, and a blind loop is created.

***The Uchida technique:*** A saline–epinephrine solution is injected into the subserosal portion of the tube, thus separating the muscular tube from the serosa. The ballooned serosa is incised and the muscular tube withdrawn. Approximately 5 cm of the tube are then excised, and the proximal end is ligated [19].

***Bipolar electrocoagulation technique:*** Bipolar forceps are used to grasp the tube, and about 3 cm of the tube are coagulated; a radiofrequency electric current is applied at three adjacent sites.

***The Hulka clip:*** is placed across the mid-isthmus. The applicator is positioned at right angles to the tube, and the entire thickness of the tube is grasped before the clip is closed.

***The Filshie clip:*** is positioned at right angles to the mid-isthmus. Viewing the posterior jaw through the mesosalpinx prior to closure ensures that the entire thickness of the tube has been grasped [15,20,21].

## 11. Post-Sterilization Regret and Symptoms of Depression

Post-sterilization regret can be assessed at various levels. Persons may be asked whether they desire (more) children, wish to reverse their previous sterilization, or by questioning women who report for sterilization reversal or in vitro fertilization (IVF). A non-representative prospective study revealed that 20.3% of women who were 30 years of age or younger, and 5.9% of those over the age of 30 years at the time of sterilization, regretted their tubal sterilization within 14 years after the procedure [2]. Tubal sterilization and the subsequent wish for reversal has been observed more frequently among black and Hispanic women than among white women [22]. Sterilization was also regretted more frequently by women with a few children at the time of sterilization, and women who wished to have children with a new partner.

A few studies have addressed the psychological impact of sterilization regret [23,24]. In a small prospective investigation conducted in Istanbul, a significant association was noted between dissatisfaction after sterilization and a higher rate of self-reported depression [25]. Studies conducted outside the scope of sterilization revealed reproductive problems as one of the most serious life stressors for women. Giving up the desire for children causes significantly greater distress for women than for men. Like women who are compelled to remain childless due to infertility, a regretted sterilization may hinder a woman’s realization of her role as a mother as well as the achievement of her life goals.

Women who undergo sterilization for reasons other than the mere desire to bear no (further) children are more likely to report regret. Women who regret their sterilization experience more severe symptoms of depression than women who do not. One study found that one of the leading risk factors for post-sterilization regret was being unmarried at the time of sterilization, and was much more common in women than men [18]. In addition to the fact that women undergo sterilization more often than men, this underlines the importance of drawing women’s attention to the permanency of sterilization and the benefits of long-acting reversible contraceptive methods. One investigation showed that approximately 70% of laparoscopic sterilizations could be performed during a one-day admission; 25% of the women experienced long-term sequelae, and 1% of the sterilizations failed [3,18].

## 12. Sterilization Reversal versus IVF

Women who desire children after tubal sterilization may opt for IVF. Tubal anastomosis is able to restore tubal function, whereas IVF is a substitute for tubal function. In consideration of fertility outcomes and costs, patients younger than 37 years were advised surgical reversal, whereas those older than 37 years of age were advised to undergo IVF. Tubal anastomosis appears to be more cost-effective than IVF in women younger than 40 years of age, whereas the opposite is true for women older than 40 years [1].

## 13. Prognostic Factors for Reversal of Sterilization

The following prognostic factors must be considered prior to surgical sterilization.

***Age*:** Age has been mentioned as the most important factor by many authors, and is reported to be inversely correlated with pregnancy rates [26,27].

***Body mass index:*** Published data in regard of BMI have been inconclusive. One study registered an impact of BMI on pregnancy rates, whereas another reported no impact [28,29].

***Postoperative tubal length:*** In four studies, the authors observed no impact of tubal length on prognosis [27,30,31,32]. However, one study revealed a higher pregnancy rate in women with longer tubes. Women who became pregnant had an average tubal length of 6.7 cm, and those who did not become pregnant had an average tubal length of 6.5 cm (*p* < 0.05) [33].

***Method of sterilization:*** Refer to the section entitled Types of Sterilization.

***Time from sterilization to reversal:*** Four studies addressed the interval between sterilization and its reversal. Three of these reported no correlation between the length of the interval and pregnancy rates [28,30,33]. One study reported a pregnancy rate of 91% at 1–5 years after sterilization, and 72% at 11–15 years after sterilization (*p* = 0.0006) [29].

***Type of anastomosis:*** The location of anastomosis and its impact on fertility outcomes was analyzed in four studies, and none of these revealed an association between the two entities [30,31,32,33].

***Ectopic pregnancies:*** We lack extensive data about prognostic factors for ectopic pregnancies. No correlation was registered between potential sites of anastomosis and percentages of ectopic pregnancies (cornual–isthmic 4.0%, cornual–ampullary 4.5%, isthmic–isthmic 2.0%, isthmic–ampullary 5.0%, and ampullary–ampullary 2.3%) [33]. Another study reported ambiguous ectopic pregnancy rates in relation to the method of sterilization. The small number of women in the Pomeroy sterilization cohort hindered an expressive statistical analysis (11% Falope ring reversal, 0% clip sterilization group, 13% coagulation, and 33% Pomeroy sterilization) [31].

## 14. Description of the Procedure

### 14.1. Docking and Instrumentation

**Figure 2:**
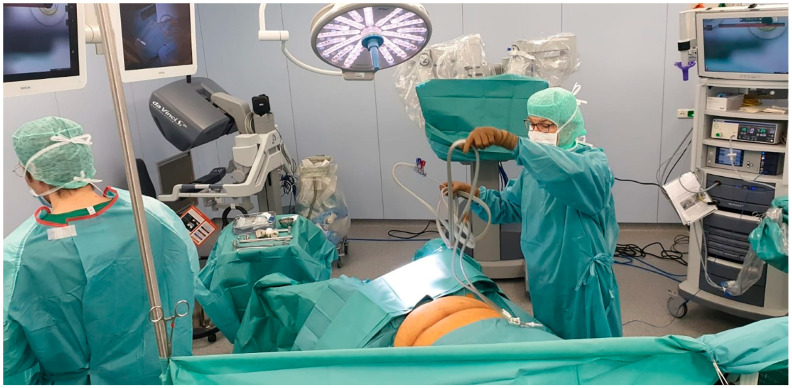
Operating room with robotic tower, Da Vinci console, patient in Trendelenburg position.

The principal operator places the robotic tower between the patient’s feet in preparation for docking (Figure 2).

**Figure 3:**
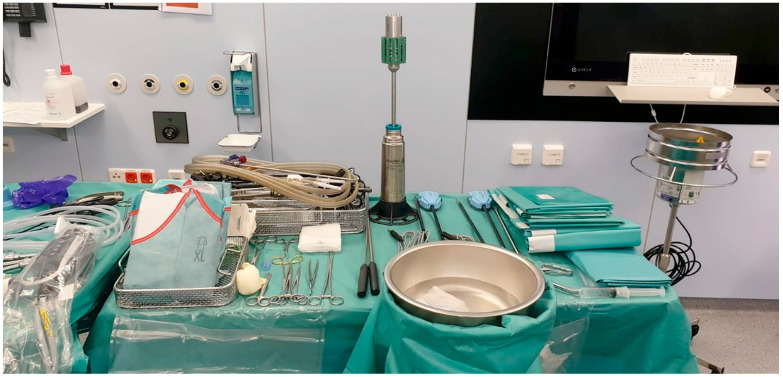
Operation settings and instruments.

The two robotic arms are attached to the lateral right and left trocars. Occasionally, a third arm is used. The robotic instruments (Figure 3) are then introduced.

### 14.2. Surgical Technique

**Figure 4:**
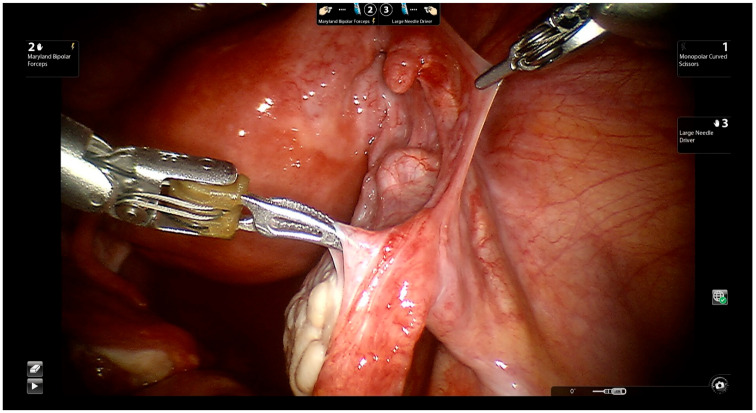
Exposing the distal and proximal ends of the fallopian tube.

The surgeon approaches the proximal and distal ends of the fallopian tube (Figure 4).

**Figure 5:**
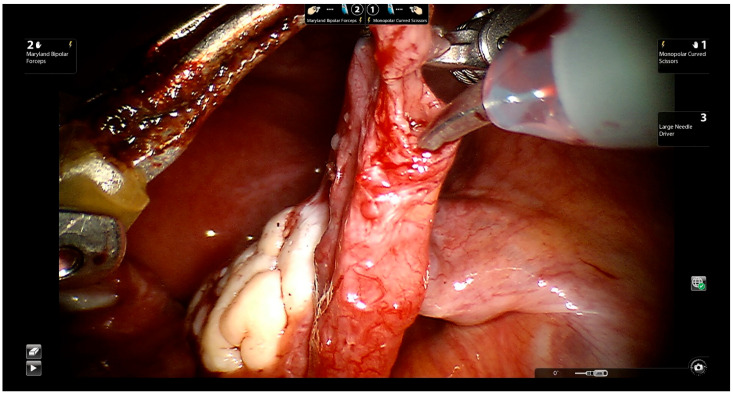
Opening the proximal and distal ends of the tube.

The surgeon injects indigo carmine dye transcervically, which permits him/her to identify the proximal portion of the tube and rule out obstruction of the proximal tube. Microscissors are then used to cut the muscularis-mucosal portion of the tube, and the proximal and distal ends are opened (Figure 5).

Reconstruction of the mesosalpinx: A series of 6-0 Vicryl stitches are performed to bridge the gap between the ends of the fallopian tube and facilitate the placement of fine sutures subsequently. The tubal segments are thus brought closer and tension on the anastomosis is prevented. If the proximal and distal anastomosis sites differ markedly in size, a stent is inserted to facilitate suturing of the two ends.

**Figure 6:**
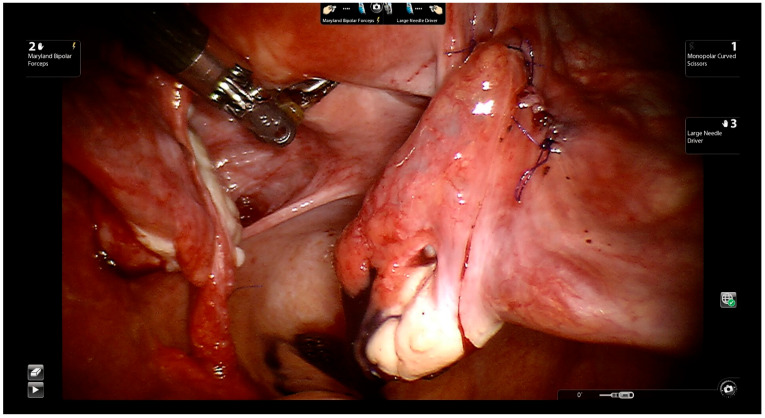
Suturing of the mucosal and muscular layers of the tubal segments with interrupted 5-0 PDS.

Tubal reanastomosis: Four to six interrupted 5-0 Vicryl sutures or PDS are used to suture the mucosal and muscular layers of the tubal segments (Figure 6).

**Figure 7:**
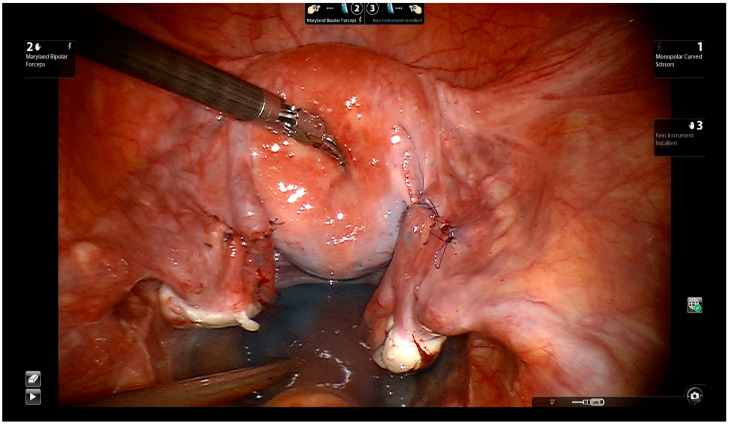
Closing the serosa with running 7-0 Vicryl sutures. Evaluating tubal patency by chromotubation.

The first suture, in the 6 o’clock position, is performed as a seromuscular knot and omits the mucosa. Three more sutures follow in order to avoid misalignment or rotation of the distal tubal segment along its longitudinal axis. As the next step, the serosa is closed separately with running 7-0 Vicryl sutures. Tubal patency and the immediate success of the procedure are confirmed by chromotubation (Figure 7, Video S1). The tubal anastomosis may be performed by mini-laparotomy, the traditional laparoscopic technique, or by Robot-assisted technology [6,7,34,35,36].

## 15. Robot-Assisted Tubal Anastomosis with the One-Stitch Technique

Robot-assisted tubal anastomosis (RATA) is a feasible and affordable method, but prolongs operating times compared to the open approach. In RATA, the tubal anastomosis is performed with multiple interrupted sutures (usually four). The one-stitch anastomosis may shorten the operating time and still achieve comparable patency and pregnancy rates. Besides, the fewer sutures may reduce the likelihood of restenosis of the fallopian tube. Further head-to-head trials will be needed to compare operating times with the multiple-stitch and the single-stitch method. RATA using the one-stitch technique for re-anastomosis appears to yield similar patency rates as RATA performed with multiple sutures [37]. 

## 16. Comparing Robot-Assisted Laparoscopic Surgery versus Conventional Laparoscopic Surgery

The numerous advantages of Robot-assisted surgery have made it increasingly popular: less blood loss, less postoperative pain, shorter hospital stays, and better visualization of fine structures [38]. Robots are used in urological, cardiac, thoracic, orthopedic, gynecological, and general surgery. In 2005, the FDA approved the use of Robot-assisted surgery in gynecology.

Robot-assisted surgery is currently being used for a variety of indications in patients with benign or malignant gynecological diseases. Telemedicine permits interdisciplinary cooperation over large distances and will, in the future, ensure comprehensive patient care by highly specialized surgery teams. The second operation console and the operation simulator constitute a new dimension in advanced surgical training. The disadvantages of Robot-assisted surgery include the high cost of the equipment and its maintenance, as well as the training of medical personnel for its competent use. In addition to these disadvantages mentioned above, Roh et al. performed a systematic review and meta-analysis and showed, in conclusion, less benefits of Robot-assisted laparoscopic surgery (RLS) compared to conventional laparoscopic surgery (CLS). The authors analyzed 1517 articles and 27 clinical reports published between 1981 and 2016 [39]. CLS showed significant advantages in total operative time, net operative time, total complication rate, and operative cost (*p* < 0.05 in all cases), whereas the estimated blood loss was less in RLS (*p* < 0.05). As a subgroup analyses, the conversion rate on colectomy and length of hospital stay on hysterectomy statistically favors RLS (*p* < 0.05) [39].

Madison et al. performed a literature review concerning surgical reversal of sterilization via reanastomosis conducted by laparotomy, conventional laparoscopy, and Robot-assisted approaches [36]. The pros and cons comparing laparotomy and conventional laparoscopy are well established and obvious, while the differences between laparoscopy and Robot-assisted approaches are more interesting. One of the biggest limiting factors in using the robot is the cost. Robots are expensive with priced ranging between USD 1 million to USD 2.5 million per unit, and often require high maintenance fees [40]. Compared with conventional laparoscopy, the Robot-assisted surgery seems to have significantly increased operative times, intraoperative complications, and a trend toward increased conversion to laparotomy. The loss of tactile feedback is discussed controversially [36]. The authors conclude that conventional laparoscopy seems to be the best approach for women < 40 years of age due to pregnancy outcomes similar to other methods, overall cost effectiveness, and the favorable safety profile of minimally invasive procedures [36].

Gynecological operations using Robot-assisted technology include myomectomy, total and supracervical hysterectomy, ovarian cystectomy, sacral colpopexy, tubal reanastomosis, lymph node dissection, surgery for retroperitoneal ectopic pregnancy, the Moskowitz procedure, and endometriosis surgery.

The difficulties faced by the anesthetist include the complex intraoperative access to the patient, the steep Trendelenburg position, the long duration of surgery, and the impact of a pneumoperitoneum. For safe Robot-assisted gynecological surgery, the surgeon must take the physiological effects of the steep Trendelenburg position and the pneumoperitoneum into account. The benefits of the surgical procedure should be weighed against its risks, especially in patients with cardiorespiratory problems.

In microscopic tubal anastomoses, the mean operating time for Robot-assisted anastomoses was significantly longer than that for open anastomoses. However, the duration of hospital stays was significantly shorter (Robot-assisted surgery 4 h; open surgery 34.7 h). After a Robot-assisted anastomosis, patients could return earlier to their activities of daily living (Robot-assisted surgery 11.1 days; open surgery 28.1 days) [41]. The study included a small number of patients, but revealed similar pregnancy rates in both groups (Robot-assisted 62.5%; open 50%). Notably, abnormal pregnancies were more common in the Robot-assisted group (ectopic: robot 4, open 1; spontaneous pregnancy loss: robot 2, open 1). The cost of delivery was similar in both groups [41,42].

Three retrospective studies and one prospective cohort study reported on the use of Robot-assisted technology for sterilization [35]. Following Robot-assisted laparoscopic surgery, pregnancy rates ranged between 50% and 70%, and the pooled pregnancy rate was 65% (95% CI: 59–72%). Only two studies provided data on ectopic pregnancy rates (11% and 22%, respectively). Collectively, the latter two studies yielded an ectopic pregnancy rate of 15%. The two-layer technique was used in two studies [35,41].

Nevertheless, there are some studies that oppositely demonstrate less prominent results in pregnancy rates of Robot-assisted techniques compared to conventional laparoscopic techniques. Goldberg et al. performed a retrospective case study and compared the length of the operation, the time until hospital discharge, the tubal patency, and the clinical pregnancy rates. The authors concluded that the operative times were 2 h longer with robotic assistance (*p* < 0.001). Furthermore, the robot provided no benefit in patient recovery and tubal patency and clinical pregnancy rates were not significantly different [43]. It is important to mention, that the number of included patients was quite small (10 patients in the Robot-assisted group and 15 patients in the laparoscopic group) and therefore, the results must be interpreted with caution.

Van Seeters et al. performed a systematic review in order to evaluate the fertility outcome of three different surgical methods available (laparotomy, laparoscopy, and Robot-assisted) for the reversal of female sterilization, compared with IVF. The authors analyzed 37 studies that investigated a total of 10,689 women. The pooled pregnancy rate after sterilization reversal was 42–69%, with heterogeneity seen from the different methods utilized. The reported ectopic pregnancy rate was 4–8%. The only prognostic factor affecting the chance of conception was female age. The surgical approach (i.e., laparotomy (microscopic), laparoscopy, or Robot-assisted) had no impact on the outcome (pregnancy rates of 68%, 65%, and 65%, respectively), with the exception of the macroscopic laparotomic technique, which had inferior results and is not currently utilized. For older women, IVF could be a more cost-effective alternative for the reversal of sterilization [1].

## 17. Pregnancy Rates in the First 12 Months after Tubal Anastomoses

One study reported a pregnancy rate of 44.3% in the first 12 months after microtubular reanastomosis (MTR). Higher pregnancy rates were noted when Hulka clips or Filshie clips were used for performing tubal occlusion, and when MTR was performed on both sides. Pregnancy rates did not differ significantly after MTR (44.3%) vs. IVF (38%). However, the authors observed a statistically significant difference in live birth rates after MTR (19%) vs. IVF (31%) (*p* = 0.007). Ectopic pregnancy and spontaneous abortion (SAB) rates after MTR were 10% and 15.7%, respectively [44,45]. Another study showed even higher pregnancy rates after Robot-assisted tubal reanastomosis. Caillet et al. performed a retrospective cohort study and included 97 patients who underwent the reversal of tubal ligation. The overall pregnancy and birth rates were 71%, (95% confidence interval [CI], 61–80%) and 62% (95% CI, 52–72%). As expected, a subgroup analysis showed lower pregnancy and birth rates with increasing maternal age [46].

## 18. Future Perspectives, Tubal Grafting

Tubal grafting or implantation could be a promising method of sterilization reversal after salpingectomy. However, extensive investigations will be needed to establish the efficacy of the method before it can be recommended for clinical use. The political and cultural acceptance of the method must also be resolved.

## 19. Conclusions

Novel technological advancements are struggling to keep pace with changes in human life. Modern lifestyles and the waning resources of our planet have altered our conceptions about the family. Families are generally planned with greater caution in western communities. In the last few years, sterilization surgery has become a common and effective method of family planning, and has been followed by advances in sterilization reversal through tubal reanastomosis. The flexibility of family planning has been enhanced by sterilization reversal. The success of reversal surgery is largely determined by age and the length of the post-sterilization interval. A third factor is the Robot-assisted approach of reversal surgery, which we consider to be superior to all others. Family planning has always been a highly sensitive and crucial issue with an enormous psychological and economic impact in the West as well as in developing countries. Therefore, any patient who wishes to undergo sterilization surgery or its reversal must be counseled comprehensively by an experienced team.

## Figures and Tables

**Table 1:** Overview of contraceptive methods.

Method	Advantage	Disadvantage	Risks	Non Contraceptive Benefits
**Coitus interruptus**	Available, free	Depends on male control	Pregnancy	Reduces risk of HIV
**Lactation**	Available, free	Duration of effect is unreliable	Pregnancy	Reduces breast cancer
**Periodic abstinence**	Available, free	Complex method, motivation is essential	pregnancy	None
**Condom**	Available, no prescription needed	Motivation is essential, must be used each time, depends on the man, skin irritation, allergic reaction	Pregnancy	Proven to reduce STDs and cervical cancer
**Spermicide and sponge**	Available, no prescription needed	Must be used each time, skin irritation, allergic reaction	Pregnancy	None
**Diaphragm, cap**	Non-hormonal	Must be used each time, fitting required	Pregnancy, cystitis	Proven to reduce STDs and cervical cancer
**Copper spiral (ParaGard** **^®^ IUD)**	Highest efficacy, unrelated to coitus	Initial cost, skilled insertion, pain and bleeding, uterine perforation, expulsion	Initial mild risk of PID and septic abortion if pregnancy occurs	None
**Levonorgestrel-releasing intrauterine system (Mirena** **^®^** **, Kyleena^®^ IUS)**	Highest efficiency, unrelated to coitus. non-contraceptive benefits: prevention of endometrial hyperplasia during menopausal hormonal therapy, endometrial protection in breast cancer patients treated with tamoxifen	Initial cost, skilled insertion, amenorrhea for some women, functional ovarian cysts, irregular bleeding (first 3–6 months), uterine perforation, expulsion	Initial mild risk of PID and septic abortion if pregnancy occurs	Reduces menstrual bleeding, can be used to treat menorrhagia
**Oral contraception, patch, vaginal ring (Nuvaring** **^®^** **)**	High efficacy	Must be taken/or changed regularly, costs	Thrombosis, risk of MI and stroke for older smokers	Can be used to treat symptoms of endometriosis and benign ovarian cyst
**Emergency contraception (levonorgestrel, ulipristal acetate)**	Moderate efficacy	Frequent use disrupts menses	None	Unknown

**Abbreviations:** STD: sexually transmitted diseases; IUD: intrauterine device; IUS: intrauterine system, PID: pelvic inflammatory disease. Derived from Berek and Novak’s Gynecology 15th edition.

## Data Availability

The datasets analyzed for the current study are available from the corresponding author on reasonable request.

## Supplementary Materials

The following supporting information can be downloaded at: https://www.mdpi.com/article/10.3390/jcm11154385/s1, Video S1: A brief video review of robotic tubal reanastomosis surgery.
